# Cortico-Cortical Evoked Potentials in Children With Tuberous Sclerosis Complex Using Stereo-Electroencephalography

**DOI:** 10.3389/fneur.2019.01093

**Published:** 2019-10-29

**Authors:** Xiaoman Yu, Ping Ding, Liu Yuan, Juncheng Zhang, Shuangshuang Liang, Shaohui Zhang, Na Liu, Shuli Liang

**Affiliations:** ^1^Department of Neurosurgery, Fourth Medical Center of People's Liberation Army (PLA) General Hospital, Beijing, China; ^2^Department of Neurosurgery, Affiliated Hospital of Jining Medical College, Jining, China; ^3^Department of Functional Neurosurgery, Beijing Children's Hospital, Capital Medical University, Beijing, China

**Keywords:** cortico-cortical evoked potential, network, preoperative evaluation, stereo-electroencephalography, tuberous sclerosis complex

## Abstract

**Objectives:** Patients with tuberous sclerosis complex (TSC) present multiple cortical tubers in the brain, which are responsible for epilepsy. It is still difficult to localize the epileptogenic tuber. The value of cortico-cortical evoked potentials (CCEPs) was assessed in epileptogenic tuber localization in patients with TSC using stereo-electroencephalography (SEEG).

**Methods:** Patients with TSC who underwent SEEG and CCEP examination in preoperative evaluation during 2014–2017 and reached postoperative seizure freedom at 1-year follow-up were enrolled in this study (*n* = 11). CCEPs were conducted by stimulating every two adjacent contacts of SEEG electrodes and recording on other contacts of SEEG electrodes in one epileptogenic tuber and its early-stage propagating tuber, and their perituberal cortexes in each patient. The CCEP was defined as positive when N1 and/or N2 wave presented, and then the occurrence rates of positive CCEPs were then compared among different tubers and perituberal regions.

**Results:** Occurrence rates of positive CCEP from epileptogenic tubers to early propagating tubers and epileptogenic tubers to perituberal cortexes were 100%, which were significantly higher than the occurrence rates of CCEP between other locations. The occurrence rates of CCEP from peripheral portions of epileptogenic tubers to peripheral portions of early propagating tubers or perituberal cortexes were 100%, which were significant higher than the occurrence rates of CCEP from peripheral regions of early propagating tubers to peripheral portions of epileptogenic tubers, from the central part of early propagating tuber to central portions of epileptogenic tubers, or from perituberal cortexes to the center part of epileptogenic tubers.

**Conclusion:** Epileptogenic tubers presented much more diffusive connectivity with other tubers and perituberal cortexes than any other connectivity relationships across propagating tubers, and the peripheral region of epileptogenic tubers presented the greatest connectivity with propagating tubers and perituberal cortexes. CCEP can be an effective tool in epileptogenic tuber localization in patients with TSC.

## Highlight

- The epileptogenic tuber demonstrated much more diffusive connectivity with other tubers and cortexes.
- The peripheral region of epileptogenic tubers presented the greatest connectivity with tubers and cortexes.
- CCEP mapping could help to confirm epileptogenic tubers.

## Introduction

Tuberous sclerosis complex (TSC) is an autosomal dominant neurocutaneous syndrome with *TSC-1/TSC-2* gene mutations (1–3). Epilepsy presented in over 90% of patients with TSC (4–6). TSC and epilepsy appear early in life and affect neurological development, with long-term effects on academic performance and, ultimately, socioeconomic outcomes. Therefore, most patients with intractable epilepsy have varying degrees of intellectual disability, which is particularly devastating among children (3, 6). However, intellectual development improvement has been related to longer periods of seizure remission (6).

Sirolimus and everolimus, inhibitors of mammalian target of rapamycin, and vigabatrin, an efficient anti-epilepsy drug for TSC-related generalized epileptic spasm, have been administrated in patients with TSC-related epilepsy, and 20–30% of those patients achieved seizure freedom, along with increased quality of life and improved behavior (4, 7, 8). Beyond drugs, vagus nerve stimulations and ketogenic diets have also been employed in patients with TSC, and 70% of those patients presented seizure reductions (9, 10). However, over 50% of patients with TSC still present intractable epilepsy under those treatments. Resective surgery is the most effective treatment for patients with TSC-related intractable epilepsy, and 53–60% of them achieve postoperative seizure freedom (11, 12).

Multiple and bilateral cortical tubers are a major character in predominant intracranial pathological changes in patients with TSC. At present, there is a unified view that cortical tubers and/or perituberal cortexes are responsible for epilepsy. Therefore, precise localization of epileptogenic tuber(s) from multiple and bilateral cortical tubers is crucial and the most difficult task in favorable postoperative seizure control in patients with TSC (4, 6, 12). High-field magnetic resonance imaging (MRI), positron emission tomography (PET), magnetoencephalography, intracranial electroencephalography (EEG), high-frequency oscillation recording, and images and EEG data post-processing have historically been utilized during preoperative assessments (13–17). Nevertheless, there still are about 50% patients with TSC who have undergone resective epileptic surgery suffering from continued seizures at 10-year follow-up after epileptic surgery (6), and wrong localization and missing epileptogenic tuber(s) are the main reasons for seizure recurrence or surgical failure. Therefore, more effective approaches to accurately localize epileptogenic tubers are important to improve postoperative seizure control.

We proposed whether epileptogenic tubers can be localized by network analyses of inter-tubers, and connections of tubers and perituberal cortexes. Effective connectivity may be empirically assessed by applying single pulses of electrical current at one cortical region and recording the cortico-cortical evoked potential (CCEP) at other remote locations (18–20). CCEP has been used to identify the brain networks and regions of functional and structural connectivity. In addition, CCEP has also been used in epileptogenic zone localization, and the ictal onset zone produces CCEPs with larger amplitudes, shorter latencies, and lower stimulation thresholds than do regions outside the ictal onset zone (21). This study aims to investigate the value of CCEP in localizing epileptogenic tuber(s) in patients with TSC-related epilepsy utilizing stereo-EEG (SEEG).

## Methods

### Patient Selection and Inclusion Criteria

Patients were enrolled and treated between August 2014 and July 2017 at Fourth Medical Center of PLA General Hospital in Beijing. All patients fulfilled the following inclusion criteria: subjects who had previously been diagnosed with TSC in accordance with the diagnostic criteria of Northrup (22); subjects who suffered drug-resistant epilepsy for over 1 year; patients who underwent SEEG examinations during preoperative evaluations (6, 23); subjects who underwent resective surgeries. Those patients who had postoperative continued seizures at 1-year follow-up or lack adequate CCEP information were excluded from the research. This study was approved by the Ethics Committee of the Fourth Medical Center of PLA General Hospital, and informed consent was obtained from all study participants.

### Stereo-Electroencephalography Examination and Epileptogenic Tuber Localization

Based on the comprehensive analyses of non-invasive preoperative evaluations, SEEG electrodes with 8–16 contacts and 0.8 mm in diameter, 2 mm in length for contacts, and 1.5 mm in inter-contact intervals (Huake Company, Beijing, China) were implanted under general anesthesia for recording intracranial EEGs to detect epileptogenic cortical tubers. The SEEG electrodes covered the cortical tubers with calcifications or cystic changes on MRI, tubers in regions with focal ictal and interictal scalp EEGs, or focal ictal symptoms, and tubers with abnormal finding on PET images, and also partial perituberal cortexes near those tubers.

Data from a minimum of three habitual seizure episodes were required for further analysis and identification. The epileptogenic tuber was identified as the first tuber with initial rhythmical discharge on SEEG before clinical seizure attack. Propagating tubers were identified by secondary rhythmical discharges on SEEG before or after clinical seizure attack in its early stage. If more than one tuber exhibited an initial rhythmical discharge on SEEG during the same seizure, or during different seizure episodes, comprehensive analyses were performed by combining data from MRI–PET co-registration, interictal high-frequency oscillation, and clinical semiology to determine which tubers were independent epileptogenic tubers and which were propagating tubers.

### Cortico-Cortical Evoked Potential Examination and Analysis

After the SEEG electrode implantation and video EEG recordings, direct cortical and subcortical electrical stimulations were performed using a 0.3-ms wide bipolar square-wave pulse with a frequency of 50 Hz for 5 s. Electrical currents were initiated at 0.5 mA and gradually increased by 0.5 mA with each train of stimulation to the patient maximum, defined as 10 mA or current at time of or after discharge or clinical sign presentation for each respective channel.

**Figure 1:**
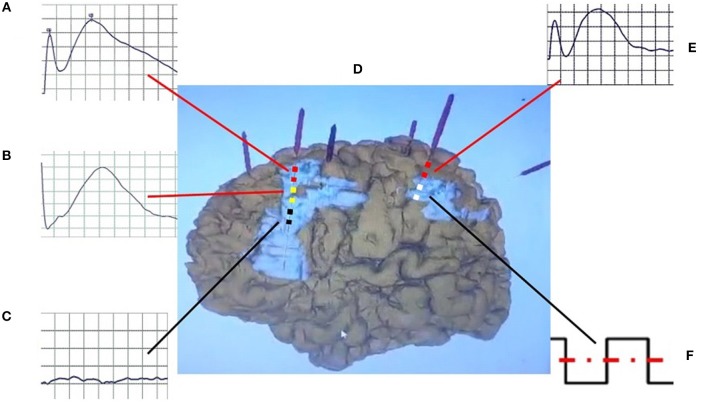
Schematic diagram of CCEP. The bipolar stimulation **(F)** was performed in the white contact of electrode in propagation tuber, and positive CCEPs were recorded on the yellow (atypical positive CCEP) **(B)** and red contacts (typical positive CCEP) **(A,E)** in the perituberal cortexes and epileptogenic tubers **(D)**, but not on the black (negative CCEP) **(C)** contacts in the center of epileptogenic tubers. CCEP, cortico-cortical evoked potential.

**Figure 2:**
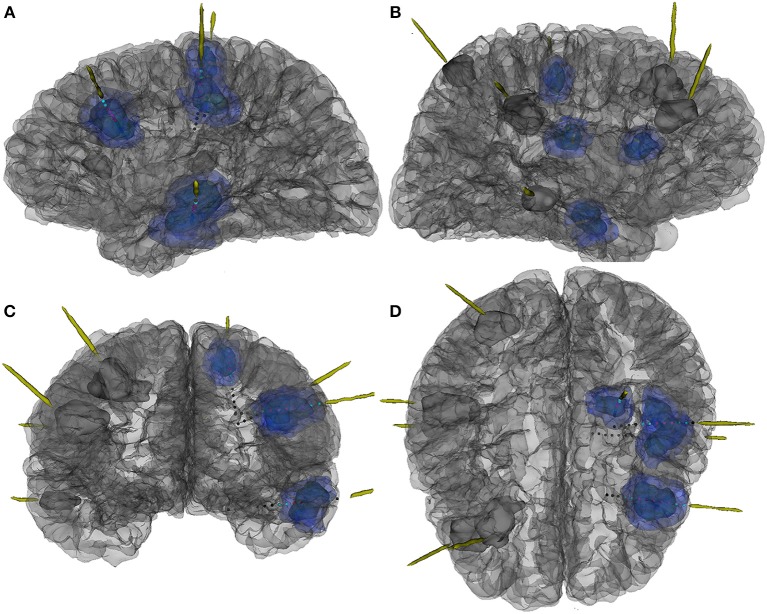
The 3D reconstruction of MR and CT fusion image to show location of electrodes and cortical tubers and their relationships in patient 7. (**A**) Left side view, (**B**) right side view, (**C**) front view, and (**D**) top view. Dark blue mass, the center part of tubers; light blue mass, the peripheral part of tubers; grey mass, the tuber without CCEP examinations; red dot, contacts in the center part of tubers; blue dot, contacts in the peripheral part of tubers; grey dot, contacts out of tubers.

CCEPs were obtained by averaging recorded signal potentials from the contacts in one epileptogenic tuber, one propagation tuber, two near non-epileptogenic tubers, and also neighboring contactors in the perituberal cortex of those tubers in each patient. The perituberal cortex was identified as cortex 10–20 mm from the boundary of a tuber covered by SEEG (Figures 1, 2). Time-locked signals were associated with the stimulation of two contacts in tubers or perituberal cortexes. The CCEP stimulations were performed with a series of 15 stimulations of bipolar square wave with pulse width of 0.3 ms and frequency of 1 Hz. The CCEP electrical currents were initiated as 80% of the maximum current and increased by 0.5 mA per train of stimulations until reaching 100% of the maximum current determined for direct cortical and subcortical stimulations.

Data were recorded from −100 to 500 ms post stimulation with a band-pass filter of 0.5–2,000 Hz. CCEP consisted of early (N1) and late (N2) negative potentials. The N1 peak was visually identified as the first small negative deflection clearly distinguishable from the stimulus artifact, and the N2 was the delayed (50–200 ms) larger negative deflection. In this study, there was obvious heterogeneity of localization of tubers and perituberal cortexes. Variability in distance inter-tuber or between tubers and perituberal cortexes, as well as stimulation currents across different sites, resulted in an inability to analyze the amplitude and latency of N1 and N2 waves. Positive CCEP was indicated by the presence of N1 and/or N2 waves at no less than one contact in tubers or perituberal cortexes during the CCEP recording when stimulations were used in any pair of contacts in other tubers or perituberal cortexes. Negative CCEP was defined as lack of N1 and N2 waves recorded with CCEP. For individuals with only N1 in CCEP, we will use another machine for repeat CCEP examination to eliminate artifacts. The technicians and doctors who performed the CCEP examinations and interpreted the results did not know which one was an epileptogenic tuber and which was a non-epileptogenic tuber.

### Statistical Analysis

Statistical analyses were completed using the SPSS statistical program (version 19.0; SPSS, Inc., Chicago, IL). Outcomes were described with percentages, means, and SD. Univariate analysis of categorical variables was performed using chi-square and Fisher's exact tests. *T*-tests and *F*-tests were used for comparison of continuous variables. When the two-tailed error probability “*P*” < 0.05, the outcome was considered to be significant. Data are presented as mean ± SD unless otherwise noted.

## Results

### Patients, Pre-surgical Evaluations, and Surgical Approach

Nineteen patients underwent SEEG electrode implantation and resective surgery between August 2014 and July 2017, and 13 of those patients reached seizure freedom at January 2019 (no <1.5 years postoperative) follow-up. At last, 11 cases (three females and eight males) were included (Table 1). Patients' average age at surgery was 5.26 ± 2.46 (range 2.6–11) years. Age of seizure onset ranged from 0.2 to 5.9 (mean 0.95 ± 1.14) years. The duration of preoperative seizures ranged from 2.3 to 9.0 (mean 4.56 ± 1.93) years. Types of clinical seizures at onset included generalized epileptic spasms (*n* = 7), focal seizure (*n* = 3), and generalized clonic seizure (*n* = 1). Seizure frequencies included either daily seizures (*n* = 9) or weekly seizures (*n* = 2). There were 6–16 (mean 10.55 ± 2.81) cortical tubers per patient (Table 2). Surgical interventions included epileptogenic tuber resections (*n* = 6), lobectomy (*n* = 3), and a combination of lobectomy and tuber resection (*n* = 2).

**Table 1:** Patients' clinical and demographic characteristics.

**No**.	**Sex**	**Age at op (years)**	**Preop course (years)**	**Age at SZ onset (years)**	**SZ type at onset**	**Number of tubers**	**Number of electrode**	**SEEG covered tubers**	**Onset tuber/propagation tuber**	**Resected tubers**	**Follow-up (years)**
1	F	4	3.8	0.2	Spasm	10	9	9	L-T-L-O L-F/L-T^*^	L-T/L-F/ LO	3.7
2	M	5.8	5.0	0.8	Spasm	16	11	10	R-F/R-FP R-T/R-TO^*^	R-F/R-FP/ R-T/R-TO	3.1
3	M	2.6	2.3	0.3	Spasm	8	7	8	R-P/R-T R-P/R-C^*^	R-P/R-T/R-C	3.0
4	F	3.8	3.3	0.5	Spasm	12	9	11	L-F/L-T/ R-F/L-F^*^ L-F/L-FP^*^	L-FP/L-T/L-F	2.7
5	F	3.7	3.4	0.3	Spasm	11	9	11	R-P/R-T R-P/R-I^*^	R-T/R-P/RI	2.2
6	M	4.3	4.0	0.3	Spasm	14	10	12	R-T/R-F	R-T/R-F	2.1
7	M	8.1	4.1	4	Focal	9	9	9	L-P/L-T	L-P/L-T	2.0
8	M	3.8	3	0.8	Clonic	8	8	8	R-T/R-FC R-F/R-I^*^	R-F/R-FC R-T/R-I	1.7
9	M	7	6.7	0.3	Spasm	11	9	10	R-F/L-F R-F/R-T^*^	R-F/L-F	1.6
10	M	6.6	5.6	1	Focal	11	9	9	L-F/L-P L-F/L-T^*^	L-F/L-P L-T	1.5
11	M	11	9	2	Focal	6	6	6	L-P/L-F L-P/L-I^*^	L-F/L-P L-I	1.5

* *This pair of onset tuber and propagation tuber was excluded from study*.

*Clonic, generalized clonic seizure; Focal, focal awareness impaired seizure; F, frontal lobe; GTCS, generalized tonic–clonic seizure; I, insular lobe; L, left; NA, not available; O, occipital lobe; Op, operation; P, parietal lobe; R, right; SEEG, stereo-electroencephalography; Spasm, generalized epileptic spasm; SZ, seizure; T, temporal lobe*.

**Table 2:**
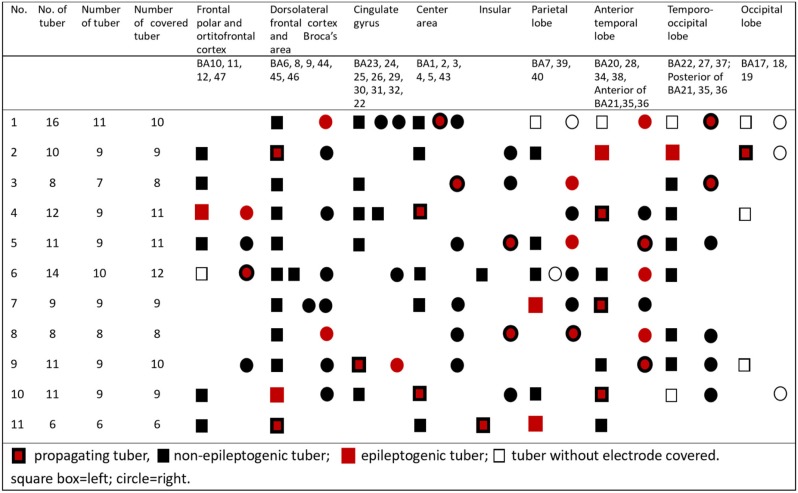
Localizations of epileptogenic tubers, non-epileptogenic tubers, and propagating tubers in all 11 patients.

### CCEP in Inter-tubers

The occurrence rate of CCEP from epileptogenic tubers to early propagating tubers was 100%, which was significantly higher (*P* < 0.05) than the occurrence rates of CCEP from early propagating tubers to epileptogenic tubers (63.6%) and from epileptogenic tubers to non-epileptogenic tubers (63.6%). The occurrence rates of CCEP from non-epileptogenic tubers to epileptogenic tubers, bidirectional CCEPs between early propagating tubers and non-epileptogenic tubers, and bidirectional CCEP inter-non-epileptogenic tubers were also significantly lower than the occurrence rate of CCEP from epileptogenic tubers to early propagating tubers (*P* < 0.01).

### CCEP Between Perituberal Cortex and Tuber

**Figure 3:**
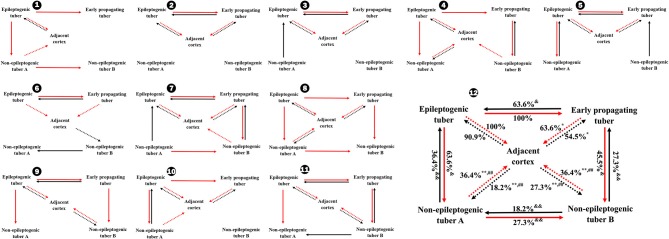
Occurrence rates of CCEP between different tubers and between tubers and perituberal cortexes in total **(12)** and individually **(1–11)**. ^*^P < 0.05, ^**^P < 0.01, the occurrence rate of CCEP in this way compared with the occurrence rate of CCEP from epileptogenic tubers to perituberal cortexes; ^##^*P* < 0.01, the occurrence rate of CCEP in this way compared with the occurrence rate of CCEP from perituberal cortexes to epileptogenic tubers. ^&^*P* < 0.05, ^&&^*P* < 0.01, the occurrence rate of CCEP in this way compared with the occurrence rate of CCEP from epileptogenic tubers to early propagating tubers. Solid line: positive CCEP inter-tuber; dotted line: positive CCEP between tuber and perituberal cortex. CCEP, cortico-cortical evoked potential.

The occurrence rate of CCEP from epileptogenic tubers to perituberal cortexes was 100%, indicative of a significantly higher CCEP occurrence rate compared with that of early propagating tubers (*P* < 0.05) and non-epileptogenic tubers (*P* < 0.01) compared with rates of perituberal cortexes. The CCEP occurrence rate from perituberal cortexes to epileptogenic tubers was 90.9%, which was significantly higher than the occurrence rate of CCEP from perituberal cortexes to non-epileptogenic tubers (*P* < 0.01) (Figure 3).

### Cortico-Cortical Evoked Potential in Epileptogenic Tubers

**Figure 4:**
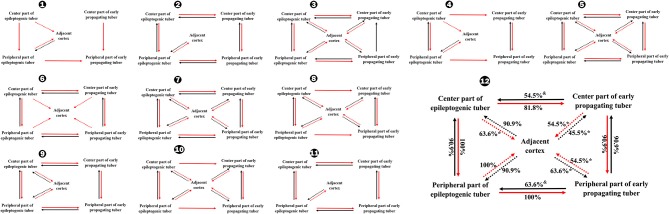
Occurrence rates of CCEP between different parts of tubers in total **(12)** and individually **(1–11)**. ^*^P < 0.05, the occurrence rate of CCEP in this way compared with the occurrence rates of CCEP from the peripheral part of epileptogenic tubers to perituberal cortexes. ^&^*P* < 0.05, the occurrence rates of CCEP in this way compared with the occurrence rates of CCEP from the center part of epileptogenic tubers to the peripheral part of epileptogenic tubers, or the occurrence rate of CCEP from the peripheral part of epileptogenic tubers to the peripheral part of early propagating tubers. Solid line: positive CCEP inter-tuber and intra-tuber; dotted line: positive CCEP between tubers and perituberal cortexes. CCEP, cortico-cortical evoked potential.

The occurrence rate of CCEP from peripheral portions of epileptogenic tubers to peripheral portions of early propagating tubers was 100%, representing a significantly higher percentage than the rate of CCEP from peripheral regions of early propagating tubers to peripheral portions of epileptogenic tubers (*P* < 0.01) and from the central part of early propagating tubers to central portions of epileptogenic tubers. Furthermore, the occurrence rate of CCEP from the peripheral part of epileptogenic tubers to perituberal cortexes was 100%, which was significantly higher than the rate of CCEP from perituberal cortexes to the center part of epileptogenic tubers, and the rates of bidirectional CCEP between central and peripheral parts of early propagating tubers and perituberal cortexes (Figure 4).

### Complications

Two seizure episodes presented during CCEP examination. Other complications were not identified.

## Discussion

To the best of our knowledge, this study represents the first comprehensive observations of CCEPs in cortical tubers of TSC patients by using SEEG. In this study, we selected the contacts of SEEG electrodes in epileptogenic tubers, propagating tubers, non-epileptogenic tubers, and perituberal cortexes as simulation and recording sites for CCEP. Observations indicated that there were connectivity with intra-tuber and inter-tubers, as well as with the perituberal cortexes, which coincides with previous reports. Kaye (24) reported a case with TSC and preserved corticospinal connectivity in a cortical tuber. The results highlighted the functional inter-digitations of tubers and eloquent cortexes. Matsumoto (20) reported connectivity between focal cortical dysplasia and perituberal cortex by CCEP. Connectivity between cortical tubers and the perituberal cortexes was also confirmed by diffusion tensor imaging (25). Nevertheless, our data demonstrate that the stimulation of epileptogenic tubers evoked 100% CCEPs in epileptogenic and perituberal tubers, which was significantly higher than the occurrence rates of CCEPs with stimulation of non-epileptogenic tubers. Our results confirmed that epileptogenic tubers demonstrate the most diffusive connectivity with other tubers and perituberal cortexes, and in turn, CCEP mapping could help identify epileptogenic tubers.

Resective range still lacks standardization in resective surgery in TSC patients regarding whether seizure originates from tubers, perituberal tissues, or both (17, 26, 27). Cortical tubers were observed to have dysmorphic cytomegalic and immature neurons, which played an important role in the generation and propagation of epileptic discharges (28, 29). Kannan et al. (26) found that focal seizures and interictal epileptiform discharges, in tuberous sclerosis, arose at the center of epileptogenic tubers and propagated into the tuber rim, perituberal cortexes, and other epileptogenic tubers. Moreover, the perituberal cortex also showed abnormal pathological changes in the gray and white matter and epileptogenicity through magnetoencephalography, electrocorticography, and intracranial EEG (27, 30–32). CCEP results confirmed that the peripheral part of epileptogenic tuber rim presented the greatest diffusive connectivity with propagating tubers and perituberal cortexes in this cohort. Therefore, the peripheral part of tuber was identified as the epileptogenic zone.

Some studies found that there were different occurrence rates and CCEP amplitudes in patients with generalized epilepsy and those with focal epilepsy (33). We did not on studying this difference, because there were multiple seizure types in patients with TSC, and most of the patients suffered generalized seizure and focal seizure at the same time. Furthermore, those multiple seizure types also varied with age and treatments, so we classified the patients by the seizure type at onset. Generalized epileptic spasm was the most common seizure at onset and presented in seven patients. However, all seven patients suffered focal seizure before surgery.

There were some limitations in this study. First, occurrence rate was the single observation factor, and the amplitude and latency of N1 and N2 waves were unsuitable for analyses owing to the heterogeneity of tuber size and locality and inter-tuber distance. Second, the exact mechanism of CCEP was not clear in that both N1 and N2 components in the CCEP were considered as oligosynaptic or polysynaptic propagation pathway (34), and positive CCEP in inter-tuber or between tubers and perituberal cortexes did not indicate that there was a direct fiber connection between epileptogenic tubers and propagating tubers or between epileptogenic tubers and perituberal cortexes. The propagating pathways from epileptogenic tubers to other tubers or cortexes need further research. Third, the perituberal cortex selections were not standardized because of limitation of the varying locations and shapes of tubers.

In conclusion, epileptogenic tubers presented much more diffusive connectivity with other tubers and cortexes than any other connectivity relationships across tuber species, and the peripheral region of epileptogenic tubers presented the greatest connectivity with tubers and cortexes. CCEP can be an effective tool in epileptogenic tuber localization.

## Data Availability Statement

The datasets generated for this study are available on request to the corresponding author.

## Ethics Statement

The study was approved by the local ethics committee and was performed in accordance with the ethical standards of the 1964 Declaration of Helsinki.

## Author Contributions

ShulL, PD, JZ, and SZ performed the operations and collected the candidates' information. XY, LY, and PD finished the CCEP examination and analyses. XY, LY, ShuaL, and NL performed EEG examination. ShulL, PD, and XY drafted the manuscript. ShulL, XY, LY, JZ, and PD analyzed datasets.

### Conflict of Interest

The authors declare that the research was conducted in the absence of any commercial or financial relationships that could be construed as a potential conflict of interest.
